# Amendment on brain signal intensity on Magnetic Resonance Imaging of asymptomatic HTLV-1 seropositive and HTLV-1 associated myelopathy

**DOI:** 10.1186/1742-4690-11-S1-P10

**Published:** 2014-01-07

**Authors:** Rafael HC Bastos, Luciana C Silva, Débora B Reiss, Gabriela S Freitas, Mariana A Souza, Cláudia LF Horiguchi, João GR Ribas, Aline M Silva, Marina L Martins, Anísia da SD Ferreira, Tatiana Martins, Anna Bárbara FC Proietti, Luiz CF Romanelli

**Affiliations:** 1 Faculdade da Saúde e Ecologia Humana (FASEH), Vespasiano, Minas Gerais, Brazil; 2 Medicina Diagnóstica ECOAR, Belo Horizonte, Minas Gerais, Brazil; 3 Grupo Interdisciplinar de Pesquisa em HTLV (GIPH), Belo Horizonte, Minas Gerais, Brazil

## 

HTLV-1-associated myelopathy (HAM) is a classic neurological disease, but cognitive impairment and altered signal intensity on MRI scans of the brain are described in the literature and suggest a more extensive neurological damage. This is a cross-sectional study, conducted between March 17^th^ and September 28^th^, 2012 in the GIPH cohort, Brazil. HAM was diagnosed on the basis of the World Health Organization diagnostic clinical criteria. MRIs of the brain axial in T1-weighted Flair, fast spin-echo T2-weighted fat-suppressed, T2-weighted Flair and diffusion weighted EPI were performed. Blinded interpretation of MRIs was performed by radiologists who did not know clinical neurologic status of the participants. Fazekas scale (0-4) was used to classify the changes in brain signal intensity. In the 120 participants the mean age of the group was 48.0 ± 12.8 years (range: 16.4 to 72.6 years), no statistically significant difference between the mean ages of the HAM and asymptomatic groups (*p* = 0.7). Women were 77 (64.2%) of patients and HAM was diagnosed in 19 (15.8%). Change of signal intensity at MRIs was observed in 48 (47%) asymptomatic group and 11 (57%) HAM group (*p* = 0.4). Both groups showed Fazekas evaluation from 0 until 3, without statistically significant differences between them (*p* > 0.05). The alteration of signal intensity on MRIs of the brain of individuals HTLV-1 seropositive asymptomatic and with HAM were not statistically significant. As a continuation of this study, we are going to compare these findings with a group of controls without HTLV-1 infection.

